# A pilot study of multilevel analysis of BDNF in paternal and maternal perinatal depression

**DOI:** 10.1007/s00737-021-01197-2

**Published:** 2022-01-06

**Authors:** Sarah Kittel-Schneider, Petra Davidova, Miriam Kalok, Corina Essel, Fadia Ben Ahmed, Yasmina Kingeter, Maria Matentzoglu, Anna Linda Leutritz, Katharina Kersken, Carolin Koreny, Heike Weber, Leonie Kollert, Rhiannon V. McNeill, Andreas Reif, Franz Bahlmann, Patricia Trautmann-Villalba

**Affiliations:** 1 Center of Mental Health, Department of Psychiatry, Psychosomatics and Psychotherapy, University Hospital, University of Würzburg, Margarete-Höppel-Platz 1, 97080 Würzburg, Germany; 2 Department of Psychiatry, Psychosomatic Medicine and Psychotherapy, University Hospital Frankfurt, Goethe University, Heinrich-Hoffmann-Str. 10, 60528 Frankfurt am Main, Germany; 3 Department of Obstetrics and Gynecology, Buergerhospital Frankfurt, Nibelungen­allee 37-41, 60318 Frankfurt am Main, Germany; 4 Institute of Peripartal Interventions, Paul-Ehrlich-Str. 10, 60596 Frankfurt am Main, Germany

**Keywords:** Paternal, Maternal, Postnatal depression, BDNF, Gene

## Abstract

Depression in the perinatal period is common in mothers worldwide. Emerging research indicates that fathers are also at risk of developing perinatal depression. However, knowledge regarding biological risk factors and pathophysiological mechanisms of perinatal depression is still scarce, particularly in fathers. It has been suggested that the neurotrophin BDNF may play a role in maternal perinatal depression; however, there is currently no data regarding paternal perinatal depression. For this pilot study, 81 expecting parents were recruited and assessed at several time points. We screened for depression using EPDS and MADRS, investigated several psychosocial variables, and took blood samples for BDNF val66met genotyping, epigenetic, and protein analysis. Between pregnancy and 12 months postpartum (pp), we found that 3.7 to 15.7% of fathers screened positive for depression, and 9.6 to 24% of mothers, with at least a twofold increased prevalence in both parents using MADRS compared with EPDS. We also identified several psychosocial factors associated with perinatal depression in both parents. The data revealed a trend that lower BDNF levels correlated with maternal depressive symptoms at 3 months pp. In the fathers, no significant correlations between BDNF and perinatal depression were found. Pregnant women demonstrated lower BDNF methylation and BDNF protein expression compared with men; however, these were found to increase postpartum. Lastly, we identified correlations between depressive symptoms and psychosocial/neurobiological factors. The data suggest that BDNF may play a role in maternal perinatal depression, but not paternal.

**Supplementary Information:**

The online version contains supplementary material available at 10.1007/s00737-021-01197-2.

## Introduction

Perinatal depression is a common disease worldwide and affects approximately 10–15% of mothers (Gavin et al. 2005). Psychosocial risk factors thought to increase the risk of developing maternal perinatal depression include previous depressive episodes, pregnancy and birth complication, traumatic birth, history of abuse, migration, low marital quality or no partner, low social status, and low social support (Biaggi et al. 2016; Anderson et al. 2017; Guintivano et al. 2018). Several studies investigating the neurobiological pathophysiological mechanisms have implicated genetic risk factors, sex hormones, and stress hormones (hypothalamus–pituitary–adrenal axis) (Meltzer-Brody et al. 2018).

In recent years, there has been increased awareness that men could also develop mental disorders that are specifically related to their partner’s pregnancy and the birth of a child. To date, several previous studies relying on screening data using the Edinburgh postnatal depression scale (EPDS) reported a prevalence of perinatal depression in fathers of approximately 5–10% (during pregnancy and up to 12 months after the birth of the child) (Massoudi et al. 2016, Johansson et al. 2017). Multiple psychosocial risk factors have been associated with an increased risk of perinatal depression in fathers, several of which overlap with risk factors for maternal perinatal depression, such as perinatal depression of the partner, low income, low social support, financial problems, stressful life events, low quality of partnership, migration background, having several children, pregnancy and birth complications (especially preterm birth) (Leung et al. 2016, Philpott and Corcoran 2018).

It has additionally been suggested that perinatal depression in fathers might negatively influence the development of their children, which is similar to the data regarding the influence of depressed mothers (Ramchandani et al. 2005; Gutierrez-Galve et al. 2019). For example, depressive fathers show reduced positive and increased negative interaction with their newborns and toddlers (Sethna et al. 2018; Miller et al. 2019). These impairments in father-child interaction were similar to those observed in depressed mothers (Wilson and Durbin 2010). Screening for perinatal depression in new fathers may therefore be equally as important as screening for pregnant women, to ameliorate the negative influence on the development of the exposed children. However, in the general public, there is still low awareness concerning the topic of perinatal mental health in fathers. Fathers and men in general also have a much higher threshold for help-seeking than mothers, especially in this period of life (Darwin et al. 2017).

In pregnant or postpartum women suffering from depression, the neurotrophin brain-derived neurotrophic factor (BDNF) has consistently been shown reduced compared to healthy controls (for a review, see (Mandolini et al. 2020)). BDNF is one of the most widely studied neurotrophins in neuropsychiatric disorders, and reduced peripheral blood concentrations have been found in schizophrenia, bipolar disorder, and major depression (Cakici et al. 2020; Schroter et al. 2020). Two studies have also investigated the association of the* BDNF* val66met gene polymorphism (rs6265) with postnatal depression in mothers. The data revealed a trend for increased risk of postnatal depression in the met allele carriers, which was more pronounced in combination with a delivery in autumn and winter (Figueira et al. 2010; Comasco et al. 2011). Furthermore, it has been shown that children exposed to maternal depression in pregnancy have increased methylation at a specific *BDNF* methylation site (CpG 5) (Braithwaite et al. 2015). However, there have been no studies investigating the potential role of BDNF in postnatal depression in fathers. To the best of our best knowledge, there is currently no data available regarding BDNF risk genotypes, epigenetic changes, and protein expression levels in paternal postnatal depression. Only a few studies have attempted to investigate potential biological mechanisms for paternal perinatal depression, and these have focused specifically on postnatal depression. These studies reported dysregulated cortisol, prolactin, and testosterone levels in fathers around birth (for a review see (Glasser and Lerner-Geva 2019)).

We aimed to investigate whether BDNF may be a marker of paternal as well as maternal perinatal depression. We hypothesized that in both perinatally depressed mothers and fathers, BDNF gene expression, gene methylation, and protein levels may be altered compared to not-depressed parents, from the period before birth until 12 months postpartum (pp).

## Experimental procedures

### Participants

For this pilot study, 86 expecting parents were recruited between July 2017 and July 2019 in the framework of the VBS study (*Vater-Bindungs-Studie*) in Frankfurt am Main (Germany). From the initially recruited couples, 81 were included in the final analysis. The couples were recruited during the monthly “expecting parent information evenings” (Bürgerhospital, Frankfurt) (for more details, see supplement). Only participants that were able to give written informed consent were included in the study, adhering to the Declaration of Helsinki (version 63, 2008). The Ethics Committee of the University of Frankfurt and the Ethics Committee of the Hesse State Medical Association (Hessische Landesärztekammer) approved the study (approval  no 135/17).

The participants were assessed during pregnancy (between 20 gestational weeks and directly before birth; T0), 3 months pp (T1), 6 months pp (T2), and 12 months pp (T3). At T0–T2, questionnaires were answered and blood samples taken for genotyping, epigenetic analysis, and protein analysis. At T3, only questionnaires were assessed. From the initially recruited couples, we obtained a full phenotypic assessment from 95% at T0, 81% at T1, 95% at T2, and 60% at T3. If our study found that participants were depressed, treatment was offered in our specialized outpatient clinic. However, data regarding treatment in our facility and/or treatment in private practice was not systematically assessed.

### Biomaterial sampling

We obtained blood samples from 87.6% participants for the first sampling (in pregnancy; T0), 83.0% at 3 months pp and 58% at 6 months pp. Blood was taken from the participants via venous puncture, usually in the evening. The participants were not fasting, as in an earlier study we found that fasting or non-fasting status did not significantly influence BDNF serum levels (Schroter et al. 2019).

### Phenotypic and demographic data

Basic demographic data was collected from all participants (age, sex, marital status, income, employment), as well as history of previous mental disorders. We collected basic self-report information regarding pregnancy and birth complications. Depressive symptoms were measured using the screening instruments Edinburgh postnatal depression scale (EPDS) (Bergant et al. 1998) and Montgomery Ǻsberg Depression Rating Scale (MǺDRS) (Schmidtke et al. 1988). As the sample was nonclinical, we used the lower cutoffs for a positive depression screening in the EPDS (≥ 10) and in the MADRS of (≥ 7) (Snaith et al. 1986; Cox et al. 1987). We also excluded sleep item analysis within the MADRS, as after birth of the infant, sleep reduction/sleep disturbances are not a reliable indicator for depression. We additionally screened for quality of partnership using the quality of marriage index (QMI) (Zimmermann et al. 2019). Furthermore, we assessed the history of childhood trauma (Childhood trauma questionnaire) (Klinitzke et al. 2012). For an overview of the demographic data see Table 1.

**Table 1:** Demographic and birth related data

	Mothers, *N* = 81	Fathers, *N* = 81	*p* value
Age in years (mean, range)	34.9 (27–43)	37.0 (28–54)	**0.014**
Working hours per week (mean h, range)	36.1 (0–55)	41.6 (0–60)	** < 0.0001**
Marital status	N (%)	N (%)	n.s
Relationship without marriage	24 (29.62)	24 (29.62)	
Married	57 (70.37)	57 (70.37)	
Living Separated	0 (0)	0 (0)	
Divorced (from a former partner)	3 (3.8)	2 (2.4)	
	3 (3.8)	2 (2.4)	
Education	N (%)	N (%)	**0.024**
No school degree	0 (0)	0 (0)	
9 years schooling	0 (0)	2 (2.4)	
10 years schooling	0 (0)	1 (1.2)	
12/13 years schooling	6 (7.4)	4 (4.8)	
Job specialization	20 (25.0)	8 (9.6)	
University degree	54 (66.6)	68 (81.9)	
Missing	1 (1.23)	0 (0)	
Income per month	N (%)	N (%)	** < 0.0001**
Information refused	6 (7.1)	5 (6.0)	
< 800 €	3 (3.7)	0 (0)	
801- 1500 €	10 (12.3)	3 (3.6)	
1501- 2000 €	11 (13.5)	3 (3.6)	
2001- 3000 €	28 (34.6)	26 (31.3)	
3001 – 5000 €	20 (24.7)	27 (32.5)	
> 5.000 €	1 (1.2)	19 (22.9)	
Missing	2 (2.5)	0 (0)	
History of mental illness	N (%)	N (%)	n.s
No	70 (97.2)	71 (94.7)	
Yes (major depression)	2 (2.8)	4 (5.3)	
Missing	9 (11.1)	6 (7.4)	
Pregnancy week at baseline visit	32.63 ± 5.45 weeks		
Breastfeeding 3 months postpartum (yes/no/not available)	53/17/11		
Subjective perception of birth	N (%)	N (%)	** < 0.0001**
		Present at birth 78 (96%)	
Neutral	5 (5.8)	3 (3.5)	
Very good	11 (12.8)	34 (39.5)	
Threatening	8 (9.3)	4 (4.7)	
Exhausting	33 (38.4)	11 (12.8)	
Overwhelming	6 (7.0)	22 (25.6)	
Missing	23 (26.7)	12 (14.0)	
Birth mode	N (%)		
Spontaneous vaginal	45 (55.5)		
Vacuum extraction	0 (0)		
Primary caesarean section	3 (3.7)		
Secondary caesarean section	14 (17.3)		
Missing	19 (23.5)		
Gender of infants male/female	N = 35/43 (41.7/52.2%)		
Missing	N = 6 (7%)		
Singletons/twins	N = 79/2		
Primipara/Multipara	N = 62/16 (76.5/19.75%)		
Missing	N = 3 (3.7%)		

Differences between men and women were calculated by Mann–Whitney* U* test or *χ*^2^ test. The level of significance was set at *p* =  < 0.05. n.s. = not significant; *N* = Number; significant *p* values are displayed in bold

### Genotyping

Genomic DNA was obtained from 150 participants (72 mothers, 78 fathers). DNA was isolated from EDTA blood by a standard procedure, as published previously (Miller et al. 1988). DNA concentration and quality were assessed by spectrophotometric measurement (Infinite 200 PRO-Tecan). Genotyping of BDNF rs6265 was performed by KASP Assay (He et al. 2014) according to manufacturer’s instructions (LGC Genomics) (see Supplemental Table 1 and Supplementary Material for more details). The distribution of the genotypes in the whole sample was 59.33% homozygous val/val, 34.66% heterozygous val/met, and 6.0% homozygous met/met. These frequencies are consistent with the reported allele frequencies in HapMap CEU (see Table 2). The observed heterozygosity of 34.66% was close to the expected heterozygosity of 33% and therefore in accordance with the Hardy–Weinberg equilibrium.

**Table 2:** Genotype frequencies in whole sample

BDNF rs6265/val66met	CC (val/val)	CT (val/met)	TT (met/met)
Women	39	30	3
Men	50	22	6
Total	89	52	9
Percentage	59.33	34.66	6.0

Genotypic distribution of BDNF val/met variant = rs6265 single nucleotide polymorphism (SNP) is given in the whole sample

### BDNF gene DNA methylation analysis

Aliquots of genomic DNA (500 ng) were bisulfite converted using the EpiTect 96 Bisulfite Kit (Qiagen) according to manufacturer’s instructions. Two amplicons covering sections of BDNF exon I promoter were amplified by PCR using the following oligonucleotides, which were specifically designed using the PyroMark Assay Design 2.0 Software (Qiagen) to amplify bisulfite converted DNA sequences (for details see Supplementary Material).

### Serum BDNF levels

A double antibody sandwich enzyme-linked immunosorbent assay (ELISA) was used to measure BDNF protein concentration in serum (DBD00; R&D Systems, USA). Protocols were conducted according to the manufacturer’s instructions and as described in our previous work (Schroter et al. 2019) (for details, see Supplementary Material).

### Statistical analysis

For statistical analysis, SPSS (Version 26, IBM, Armonk, NY, USA) was used. The graphs were created using GraphPad Prism (Version 9, GraphPad Software, San Diego, CA, USA). Data were tested for normal distribution. Parametric tests (ANOVA tests, Student’s *t* tests, Pearson’s correlation, Chi-square test) or nonparametric tests (Mann–Whitney *U* test, Kruskal–Wallis test, Spearman rho correlation, Kendell-tau test) were performed accordingly. Due to the exploratory nature of the study, we did not correct for multiple comparisons for most data, and set the level of significance at *p* = 0.05. However, we conducted a Bonferroni correction for the gene methylation results, as we tested for 14 CpG sites. The level of significance was therefore set at *p* = 0.004. A post hoc power analysis of the results of the BDNF protein levels and depressive symptoms revealed a sufficient power of 0.8.

## Results

### Perinatal depression prevalence and associated psychosocial risk factors

In our baseline visit during pregnancy (T0), we found that 3.7% men and 11.25% women showed elevated depressive symptoms using the EPDS screening instrument and a cutoff of ≥ 10. These results were validated by MADRS interview. Moreover, MADRS showed increased numbers of participants with scores ≥ 7, indicating mild depression. Using the MADRS, 7.4% men and 21.25% women showed mild depressive symptoms at T0. When we subtracted the MADRS item assessing sleep, the results for fathers did not change. However, only 13.8% mothers still had depressive symptoms. MADRS and EPDS scores significantly positively correlated at all measured time points in the whole sample (*p* ≤ 0.003 for every time point). Three months postpartum, 5.6% fathers and 20.9% mothers showed elevated depressive symptoms in the EPDS, and 16.4% fathers and 21.9% mothers showed elevated depressive symptoms using the MADRS (minus the sleep item). Using the total MADRS score (with sleep item), the percentage of fathers with elevated depressive symptoms was marginally higher at 17.8%, whereas 41% of mothers scored ≥ 7 in the MADRS.

**Fig. 1:**
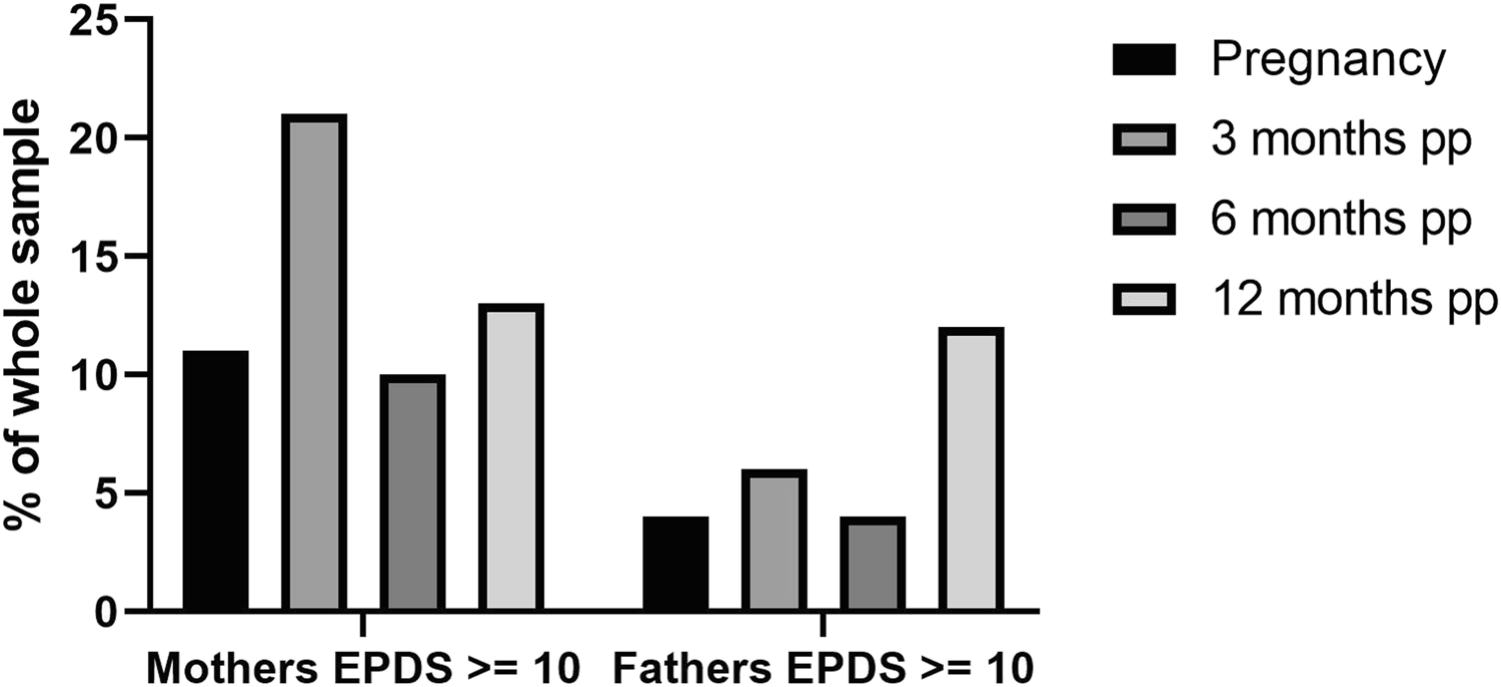
Proportion of depressed parents (EPDS). The proportion of mothers and fathers that scored ≥ 10 in the EPDS is shown here as a percentage of the whole sample from pregnancy until 12 months postpartum. PP = postpartum

**Fig. 2:**
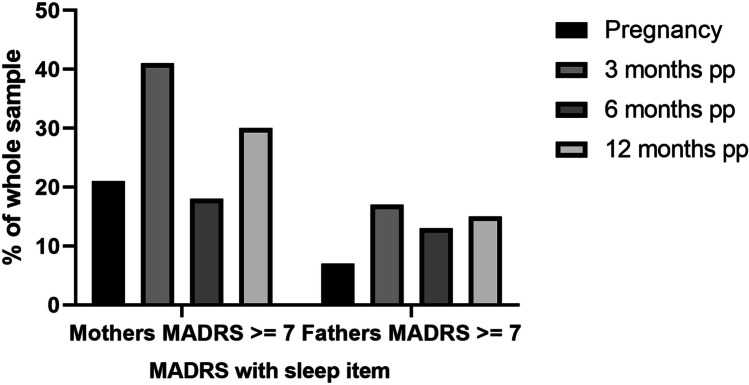
Proportion of depressed parents (MADRS total score). The proportion of mothers and fathers that scored ≥ 7 in the MADRS is shown here as percentage of the whole sample from pregnancy until 12 months postpartum. PP = postpartum

**Fig. 3:**
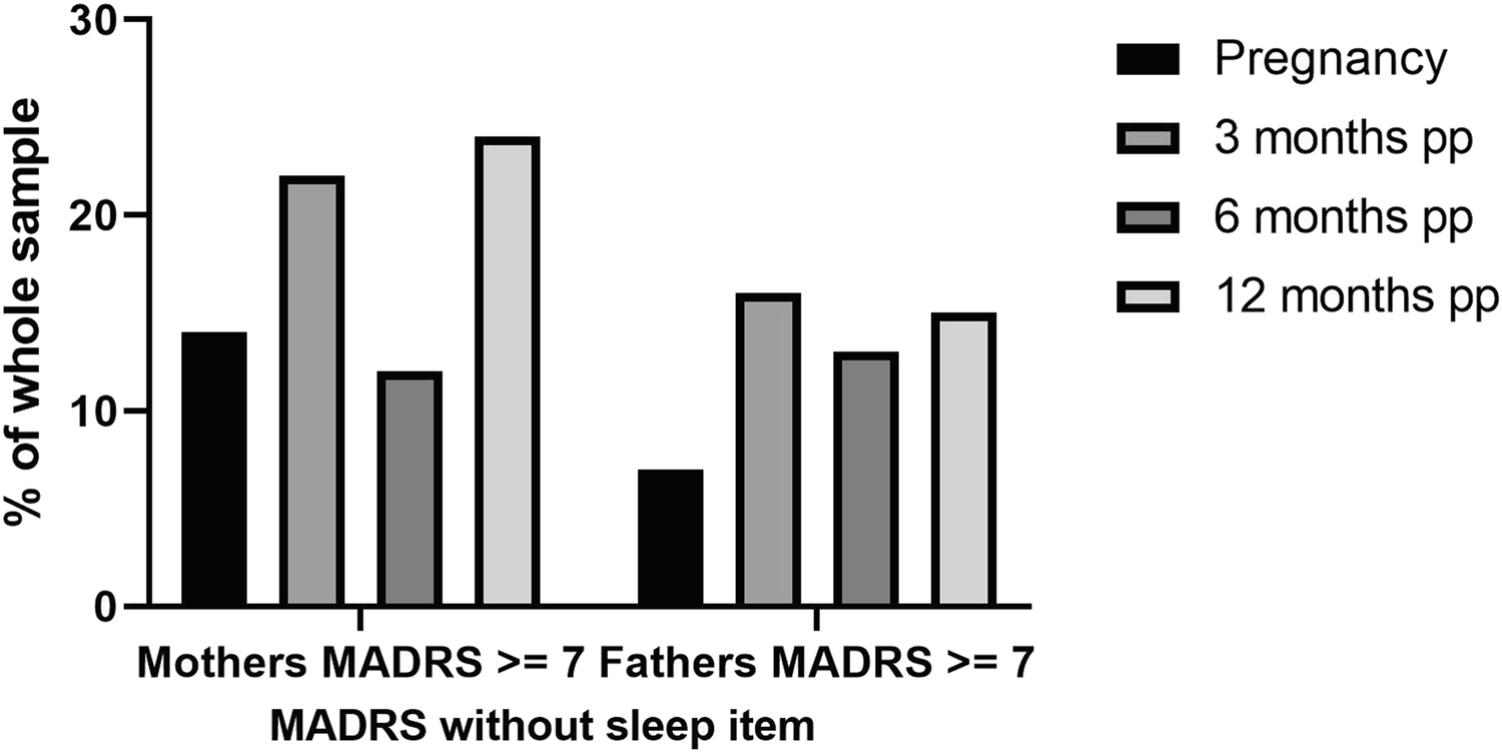
Proportion of depressed parents (MADRS without sleep item). The proportion of mothers and fathers that scored ≥ 7 in the MADRS but without the sleep item is shown here as percentage of the whole sample from pregnancy until 12 months postpartum. PP = postpartum

Six months postpartum (pp), the prevalence of elevated depressive symptoms decreased to 3.6% in fathers and 9.6% in mothers using the EPDS and 13.1% (fathers) and 18% (mothers) using the total MADRS score. The percentage of fathers with depressive symptoms did not change after excluding the sleep item; however, the percentage decreased to 12% in mothers upon sleep item exclusion. Interestingly, the rate of fathers that scored positive for depression using the EPDS increased after 12 months postpartum to 12%, and 15.7% screened positive using the MADRS at this time point (with and without the sleep item). 13.5% mothers had depressive symptoms using the EPDS at 12 months postpartum; however, this increased to 30% and 24% in the MADRS with and without the sleep item, respectively (see Figs. 1, 2 and 3 and Supplemental Figs. 1–3). Most participants only experienced mild depression (for depression severity ratings using MADRS see Supplemental Tables 1 and 2). Using either the EPDS or MADRS, 25.6% fathers screened positive for depression at least once during the study period, and 48.1% mothers. 69.2% fathers who screened positive for depression experienced the increased depressive symptoms during pregnancy and the first 3 months postpartum for the first time, 15.4% between 3 and 6 months, and 15.4% between 6 and 12 months postpartum. There was no significant correlation between pregnancy week and depressive symptoms in either the EPDS or MADRS with/without sleep item (all *p* > 0.5).

MADRS scores both with and without the sleep item were significantly correlated between women and men, suggesting that perinatal depression of one partner is likely to also affect the other partner (Spearman rho correlation, all p < 0.007). However, EPDS scores were not correlated between women and men, even though MADRS and EPDS scores were significantly positively correlated in the whole sample (Spearman rho correlation, all *p* < 0.0001).

Regarding risk factors, fathers with a history of depression were significantly more likely to display depressive symptoms during the pregnancy (*χ*^2^ test, *p* = 0.007). However, this result was not significant for women. Participants with a lower income had a higher rate of postnatal depression at 3 and 12 months postpartum (*χ*^2^ test, *p* = 0.008; *p* = 0.002). Women who were unemployed were significantly more likely to report elevated depressive symptoms during pregnancy (*χ*^2^ test, *p* < 0.0001), as were unemployed men (χ^2^ test, *p* = 0.013). Quality of marriage, assessed by the QMI screening questionnaire, was significantly correlated with depressive symptoms measured by the EPDS. QMI measures were significantly negatively correlated with EPDS scores at all time points in fathers, and during pregnancy and 12 months postpartum in mothers, suggesting an association between worse relationship quality and depressive symptoms (Table 3). The sex of the baby was not significantly associated with depression in either the mother or the father (*χ*^2^ test all *p* ≥ 0.1). There was no significant difference in depressive symptoms between mothers who were breastfeeding at 3 months postpartum and those who were not breastfeeding (Mann–Whitney *U* test, all *p* > 0.48).

**Table 3:** Correlation of marriage quality and depressive symptoms (EPDS)

Visit	QMI, men (mean)	QMI, women (mean)	EPDS, men (mean)	EPDS, women (mean)	Correlation with EPDS
t0	41.2	41.8	3.11	4.51	M: *r* = − 0.361, *p* < 0.001 F: *r* = − 0.313, *p* = 0.006
t1	40.5	39.8	3.14	5.31	M: *r* = − 0.265, *p* = 0.026 F: n.s
t2	40.2	30.8	3.11	4.18	M: *r* = − 0.265, *p* = 0.031 F: n.s
t3	38.6	36.6	2.88	4.62	M: *r* = − 0.530, *p* < 0.001 F: *r* = − 0.395, *p* = 0.004

The sum scores of QMI and EPDS were correlated in men and women in all study visits (t0 = pregnancy, t1 = 3 months postpartum, t2 = 6 months postpartum, t3 = 12 months postpartum). Spearman rho correlation was measured, level of significance was set at *p* ≤ 0.05

QMI = quality of marriage index; EPDS = Edinburgh postnatal depression scale; M = male, F = female

Childhood trauma is a known risk factor for depression, and pregnancy/birth of a child can also trigger own trauma experiences of the parents, as well as being perceived as a traumatic experience itself. Moreover, traumatic experiences have been shown to influence gene methylation. We therefore included the childhood trauma questionnaire (CTQ) in our analysis. In our sample, women reported marginal significantly higher values for emotional abuse (Mann–Whitney *U* test *p* = 0.05), whereas reported values for physical abuse, physical neglect, sexual abuse and emotional neglect were similar between men and women (Mann–Whitney *U* tests all *p* > 0.05). Men with elevated depressive symptoms at 3 months postpartum (MADRS with/without sleep item) had significantly higher values for physical neglect compared to men that screened negative for depression at both baseline (Mann–Whitney *U* tests *p* = 0.004) and after 3 months (Mann–Whitney *U* tests *p* = 0.003). Men with elevated depressive symptoms at 3 months postpartum additionally reported significantly higher values for physical abuse at 12 months postpartum compared to nondepressed men (Mann–Whitney *U* tests *p* = 0.026). There was a significantly higher score for physical neglect in childhood in women with increased depressive symptoms (according to MADRS score without sleep item) at baseline (in pregnancy) and after 12 months (Mann–Whitney *U* test, *p* = 0.039; *p* = 0.036) and for emotional and physical abuse in women with increased depressive symptoms 12 months postpartum (Mann–Whitney U test, *p* = 0.04, *p* = 0.045).

### BDNF gene variant val66met

There were no significant differences in depressive symptoms (EPDS/MADRS) between the BDNF val/val, val/met, and met/met genotypes, neither in the whole sample nor when the mothers and fathers were analysed separately (Kruskal–Wallis test; Kendell-tau-b test, all *p* > 0.05).

### BDNF gene methylation

**Fig. 4:**
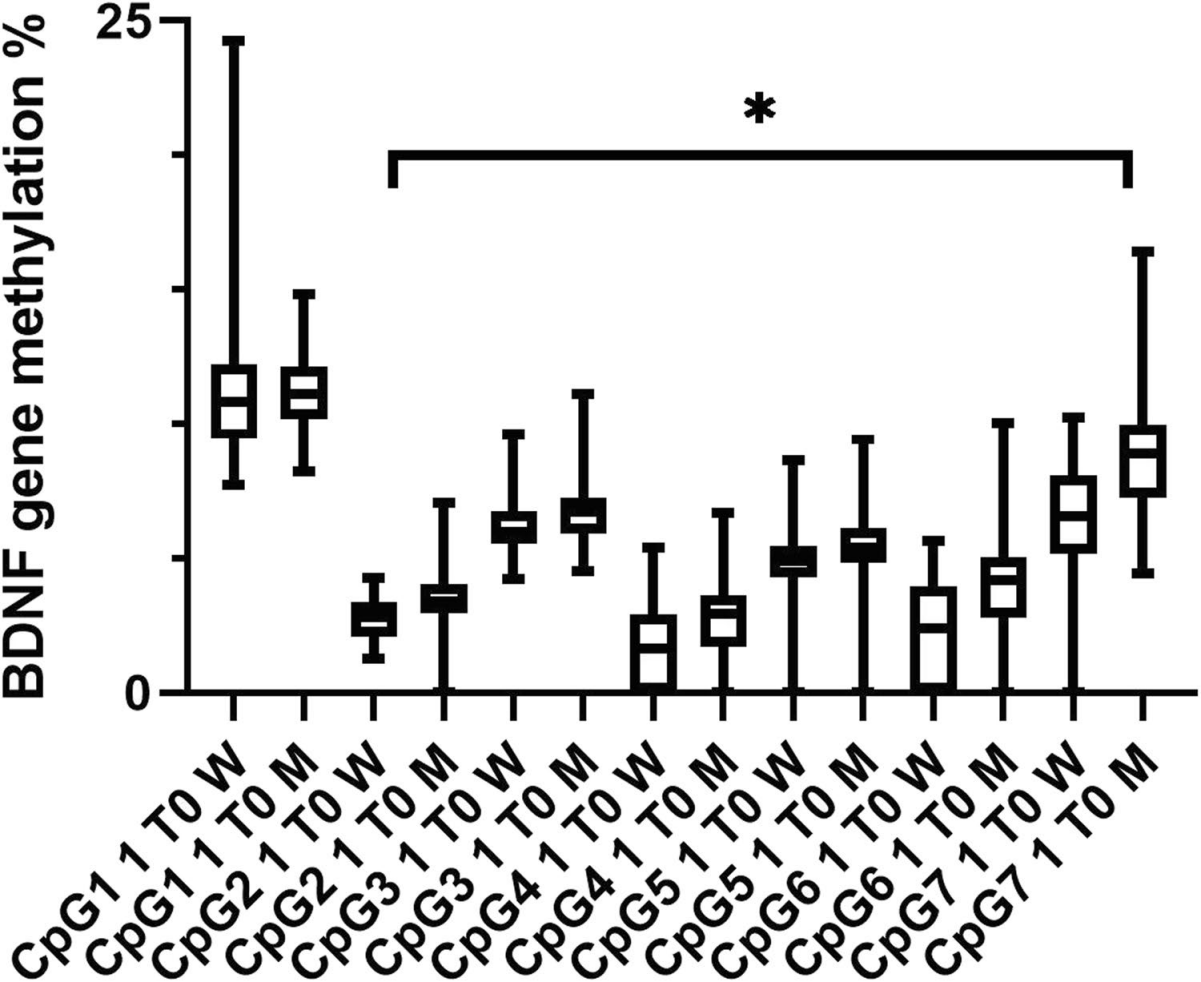
BDNF gene methylation in sequence 1 between pregnant women and men. Mean percentages of BDNF gene methylation are shown here ± standard deviation. Except in CpG site 1, in all other CpG sites men showed higher methylation levels than pregnant women (Mann–Whitney *U* tests, all *p* < 0.004). CpG = ; T0 = baseline visit in pregnancy; W = women; M = men. Level of significance was set at *p* ≤ 0.004. * *p* ≤ 0.004

**Fig. 5:**
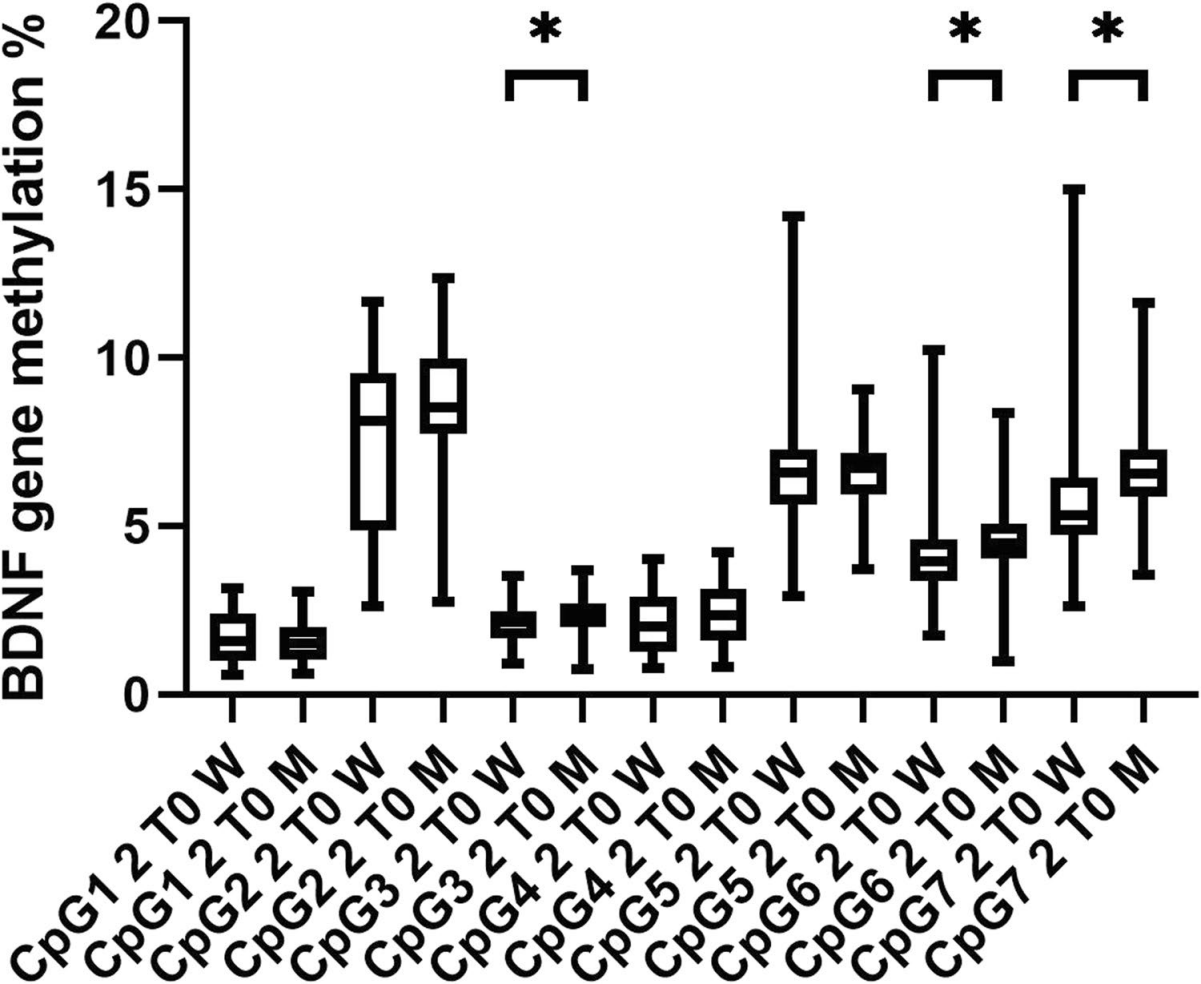
BDNF gene methylation in sequence 2 between pregnant women and men. Mean percentages of BDNF gene methylation are shown here ± standard deviation. In CpG sites 3, 6, and 7 men showed significant higher methylation levels than pregnant women (Mann–Whitney *U* tests, all *p* < 0.004). CpG = ; T0 = baseline visit in pregnancy; W = women; M = men. Level of significance was set at *p* ≤ 0.004. * *p* ≤ 0.004

Pregnant women had significantly lower BDNF gene methylation than men in CpG sites 2, 3, 4, 5, 6, and 7 of sequence 1, and CpG sites 6 and 7 of sequence 2, even after correction for multiple comparisons (Mann–Whitney *U* test, all *p* ≤ 0.004) (see Figs. 4 and 5). Additionally, pregnancy week was significantly positively correlated with methylation at CpG site 2 of sequence 2 (Spearman’s *r* = 0.55, *p* < 0.0001). At 3 and 6 months postpartum, there were no longer any significant differences between women and men (Mann–Whitney *U* test, all *p* ≥ 0.03). No significant differences in BDNF gene methylation were observed when comparing breastfeeding and non-breastfeeding women at 3 month pp (Mann–Whitney* U* test, all *p* > 0.16).

We did not find any significant differences in BDNF methylation level between participants who screened positive for depression and those who screened negative using the EPDS and/or the MADRS (with/without sleep item) scores (Mann–Whitney *U* test, all *p* > 0.004) when analysing the whole sample. After correction for multiple testing, there was no significant negative correlation between the EPDS or MADRS (with/without sleep item) and methylation of any of the CpG sites (all *p* > 0.004). In the sex-separated analysis, there was no correlation that withstood correction for multiple testing, in either the mothers (Spearman rho correlation, all *p* > 0.004) or the fathers (Spearman rho correlation, all *p* > 0.004). Additionally, correlations between the subdomains of the childhood trauma questionnaire and BDNF methylation were no longer significant after correction for multiple testing, in mothers or in fathers (Spearman rho correlation, all *p* > 0.005).

### BDNF protein levels

Between the *BDNF* val66met genotypes there was a marginally significant difference in BDNF protein levels in the whole sample. TT carriers had a significantly higher protein concentration at T0 (Kruskal–Wallis test, *p* = 0.043) compared with CT and CC carriers, whereas there only a trend for increased protein concentration when comparing CC to CT/TT carriers, (Mann–Whitney *U* test, *p* = 0.084). At T0, there was a significant positive correlation between BDNF gene methylation at sequence 1 CpG site 2 and BDNF protein concentration in the whole sample (Spearman rho correlation, *r* = 0.285, corrected *p* = 0.001).

BDNF protein levels were not significantly different between participants who screened positive for depressive symptoms (EPDS/MADRS) and those who screened negative in the whole sample. There were also no significant differences when the sexes were analysed separately.

**Fig. 6:**
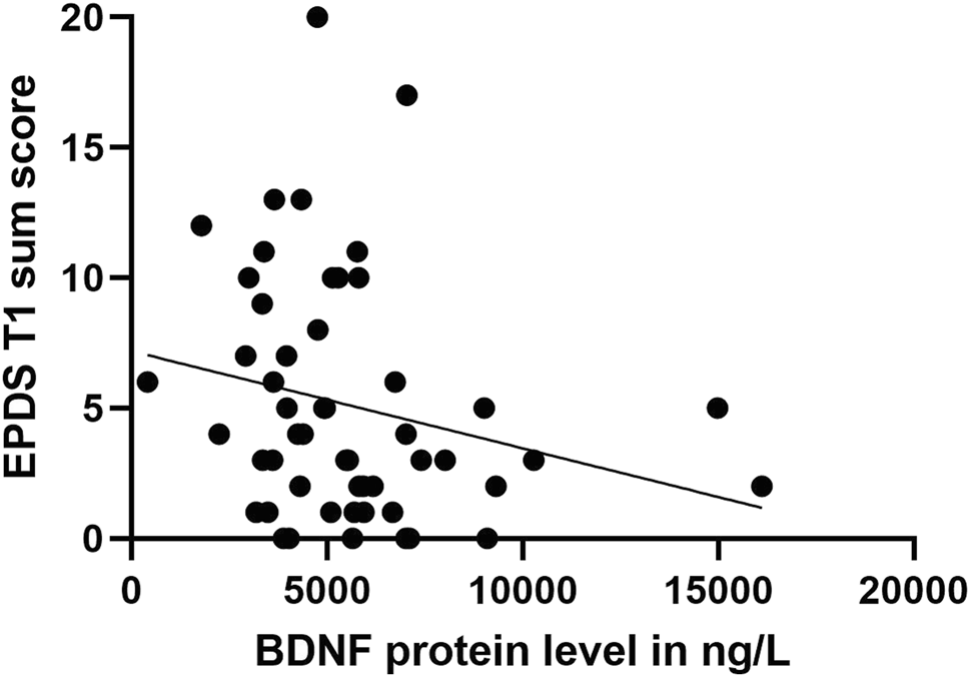
Correlation of depressive symptom with BDNF protein levels in mothers at T1. Correlation of mean levels of BDNF serum concentration and mean EPDS sum scores 3 months pp in mothers is shown. There was a significant negative correlation (Spearman rho correlation − .307, *p* = 0.027)

EPDS/MADRS scores and BDNF protein levels were not found to be significantly correlated in the whole sample (Mann–Whitney *U* tests, all *p* ≥ 0.05). However, EPDS scores and BDNF protein concentration were significantly negatively correlated at 3 months postpartum in women (Spearman rho correlation *r* =  − 0.307, *p* = 0.027) (Fig. 6). This negative correlation was also found when using MADRS with/without sleep item (Spearman rho correlation *r* =  − 0.275, p = 0.04; *r* =  − 0.325, *p* = 0.015, respectively). Surprisingly, there was a significant positive correlation between the EPDS score and BDNF protein levels at T0 in the expecting fathers (Spearman rho correlation, *r* = 0.252, *p* = 0.03); however, this correlation could not be replicated when using the MADRS scores with/without sleep item (Spearman rho correlation, all *p* >  = 0.05).

In the whole sample, pregnant women had significantly lower BDNF protein concentrations compared with men (Mann–Whitney *U* test, *p* < 0.0001). However, at 3 months postpartum, BDNF protein concentration was significantly higher in women than in men (Mann–Whitney *U* test, *p* = 0.024). There was no significant correlation between pregnancy week and BDNF protein concentration (Spearman correlation, *r* = 0.06; *p* = 0.7). There were no significant sex-differences in BDNF protein concentration at 6 months postpartum (Mann–Whitney *U* test, *p* = 0.156) (see Supplemental Fig. 5). No significant differences in BDNF protein concentration were found between mothers who were breastfeeding at 3 months postpartum and those who were not breastfeeding (Mann–Whitney* U* test, *p* > 0.24).

## Discussion

The aim of this pilot study was to investigate the prevalence of maternal and paternal perinatal depression and additionally identify psychosocial and biological risk factors. Despite a relatively small sample size, we found that fathers are also at risk of developing peri- and postnatal depression, consistent with previous studies. One previous meta-analysis reported an incidence of ~ 8.4% for paternal perinatal depression (Cameron et al. 2016), and a more recent meta-analysis of observational surveys including 20,728 participants found a paternal prenatal depression prevalence of 9.76% in all three trimesters (Rao et al. 2020). Specifically, the prevalence of postpartum depression in fathers was found to be 7.82% during the first 3 months, which is higher than our results using the EPDS (3.7%) but consistent with our MADRS screening results (7.4%). The authors also reported a prevalence rate of 9.23% between the third and sixth month postpartum and 8.4% between 6 and 12 months. These incidence rates are lower than our data using the MADRS without the sleep item (3 months pp: 16.4%; 6 months pp 13.1%; 12 months pp 15.7%) but higher than our data using the EPDS, except at 12 months (3 months pp: 5.6%; 6 months pp 3.6%; 12 months pp 12.0%) (Rao et al. 2020). Interestingly, the highest depression rates in fathers were found at 3 months postpartum and 12 months postpartum in our sample, a result which was also replicated in the mothers (depression prevalence rate in mothers was 21% (EPDS)/21.9% (MADRS without sleep item) after 3 months pp and 13.5% (EPDS)/24% (MADRS without sleep item) after 12 months pp. A study of Italian fathers reported a similar time course, with depressive symptom incidence rate increasing in first time fathers 12 months after birth of the child (Molgora et al. 2017). The rates of perinatal depression in our mothers are also consistent with previous studies (Buist et al. 2008).

Several psychosocial and medical risk factors have been identified as associated with the risk of postnatal depression in mothers and fathers, such as low socioeconomic status (Cena et al. 2021), preterm birth (de Paula Eduardo et al. 2019), low social support, immigration background (Anderson et al. 2017), violence and abuse, gestational diabetes, cesarean section, history of depressive episodes, multiple births, negative birth experience, postnatal anemia, vitamin D deficiency, obese and overweight, and postpartum sleep problems (for a review regarding mothers see (Zhao and Zhang 2020) and for fathers (Edward et al. 2015)). In our study, we could confirm that both lower socioeconomic status of parents and previous depressive episodes in fathers were associated with increased perinatal depressive symptoms. We additionally found a decrease in relationship quality after birth and that a lower relationship quality significantly correlated with depressive symptoms in both mothers and fathers, consistent with the results of a previous Swedish study (Johansson et al. 2017). As there were no preterm children or severe pregnancy and birth complications in our sample, we could not investigate these factors.

Despite our small sample size, we found a positive correlation between maternal and paternal depressive symptoms, consistent with a previous meta-analysis including 28,004 participants. However, the authors report that this result was only significant for MADRS values, and not EPDS values (Paulson and Bazemore 2010). Previous studies have suggested that the EPDS may be more suitable for screening for worry, anxiety, and unhappiness in fathers, rather than depression, which might partially explain why we did not find a correlation between depressive symptoms in the parents from our EPDS data (Massoudi et al. 2013). The percentage of women that showed depressive symptoms was also higher measured by MADRS with and without sleep item compared to measurements using the EPDS. As sleep problems are very common in pregnancy, and sleep is naturally disturbed after the birth of a child, this explains the much higher scores in maternal MADRS with sleep item included (Bei et al. 2015). However, even when removing the sleep item from the total score, the number of mothers with depressive symptoms was higher using the MADRS than the EPDS. A possible reason could be that the EPDS does not assess appetite, concentration difficulties, or reduced drive, which all could be affected by pregnancy and in the postpartum period, without necessarily being caused by depression (Brown and Schaffir 2019, Baskin et al. 2021). Despite these subtle differences in EPDS and MADRS scores, the two screening methods showed a significant positive correlation in the whole sample. Therefore, both screening instruments appear to measure similar psychopathology, or at least have a large overlap.

BDNF is one of the most studied neurotrophins in mental disorders; therefore, we investigated its potential role as a biological risk factor for perinatal depression. In the whole sample, there was only a marginally significant difference in BDNF serum concentrations between the different BDNF val66met genotypes, with TT carriers showing the highest BDNF concentration at T0. However, this result could have been influenced by sex, as most TT carriers were male (6 vs. 3 females, see Table 2) and women were found to have significantly reduced BDNF levels in pregnancy. There were no significant differences in depressive symptoms between the rs6265 genotypes; however, this could be due to the small sample size available for genetic analysis.

We next investigated BDNF gene methylation. Pregnant women showed decreased gene methylation in eight of the fourteen investigated CpG sites in the BDNF gene. In a previous study, we found evidence for a positive correlation between BDNF gene methylation level and BDNF protein levels at CpG sites 1 and 6 in a sample consisting of bipolar and unipolar depression patients and healthy controls (Schroter et al. 2019). This is consistent with our current findings, which demonstrated a positive correlation between BDNF methylation in sequence 1 CpG sites 2, 4, 5, 6, and 7 with BDNF protein level at T0 in the whole sample. However, these results did not remain significant after correction for multiple comparisons. Gene hypermethylation often leads to decreased gene and protein expression; however, this effect is location-dependent. The amplicon we investigated contains seven CpG sites in each sequence, located within the promoter region of the BDNF gene, and covers a highly active transcriptional region. Methylation patterns of this BDNF gene region have not yet been investigated regarding functional consequences on gene expression and protein levels. However, epigenetic modifications of a gene promoter region are known to regulate gene expression. Hypomethylation generally leads to increased mRNA levels and consequently increased protein expression, whereas hypermethylation of the gene promoter usually represses gene expression (Suzuki and Bird 2008). However, hypomethylation can also result in decreased gene and protein expression, which could have led to the decreased BDNF gene and protein expression observed in our study.

We next investigated a potential correlation between depressive symptoms, traumatic experiences in childhood (measured by CTQ), and *BDNF* methylation. Altered *BDNF* methylation has previously been associated with childhood abuse in patients with borderline personality disorder and eating disorders (Perroud et al. 2013, Thaler et al. 2014). A systematic review also found BDNF gene methylation changes associated with childhood trauma (Nothling et al. 2020). The role of altered *BDN*F gene methylation in major depression has also been recently reviewed, and the data found consistent (Li et al. 2019). After correction for multiple comparisons, we did not find a significant correlation between hypomethylation in different CpG sites and depressive symptoms in our sample nor increased history of childhood abuse or neglect. We were therefore unable to replicate a correlation between altered *BDNF* methylation and childhood trauma/depression. However, this may be because we had a relatively small sample size of depressed participants, who were mostly only mildly to moderately depressed and not a clinical sample as in previous studies.

BDNF protein levels have previously been studied in maternal but not paternal perinatal depression (Gao et al. 2016). A meta-analysis reported lower BDNF serum levels in maternal perinatal depression (Mandolini et al. 2020), which is partly consistent with our finding of a significant negative correlation between depressive symptoms and BDNF protein levels in mothers. Previous studies specifically reported an association of lower BDNF levels and later antepartum depression in early pregnancy, but no correlation with depression severity (Fung et al. 2015). Furthermore, decreased BDNF concentration in late pregnancy has been associated with increased risk of depression (Christian et al. 2016), and lower BDNF concentration has been reported in mothers with PPD compared to mothers without depressive symptoms (Gazal et al. 2012). Gao et al. also suggested that a BDNF protein concentration < 12 ng/ml could be a predictive marker of PPD (Gao et al. 2016). However, in our German sample, most participants had a BDNF concentration lower than 12 ng/ml. However, this could have been due to different assays used; therefore, the standardization of analysis methods would be a prerequisite to determine feasibility of translation into clinical routine diagnostics.

In our male participants, a significant positive correlation between EPDS score and BDNF concentration was found at the baseline visit. However, variance in the BDNF measurements was large. Moreover, there are several extraneous variables that are known to influence BDNF concentration, such as physical activity (Walsh et al. 2020). We did not assess the quality and quantity of physical activity in our pilot study, but it is likely that there are unknown confounders influencing the positive correlation between increased BDNF concentration and depressive symptoms in our male participants.

Previous studies have shown lower BDNF protein concentrations in pregnant women (Christian et al. 2016). However, a recent study investigated depressed vs. remitted depressed vs. nondepressed pregnant and nonpregnant control women, with a follow-up at 6 weeks postpartum. The authors reported a higher BDNF plasma concentration in the pregnant group compared with nonpregnant controls and a lower concentration in the postpartum depression group compared with the nondepressed group. In the women that had already recovered from postpartum depression, BDNF concentration increased at 6 weeks after delivery compared to levels during pregnancy. The reason for pregnancy-related changes in BDNF concentration currently remains unclear (Lee et al. 2021). We could partially validate previous findings in our sample, as pregnant women showed significantly lower BDNF protein concentrations than their male counterparts. At 3 and 6 months postpartum, BDNF protein concentration increased in the mothers. At 3 months postpartum, BDNF protein concentrations were higher in women compared with men, and at 6 months concentrations were similar between the sexes.

Dysregulation of neurotrophins such as BDNF may lead to negative effects on maternal and fetal health (for a review, see (D'Angelo et al. 2020)). Maternal exposure to several toxins such as ethanol, smoking, and illegal drugs reduce BDNF in the fetal brain, leading to impaired neurodevelopment (Carito et al. 2019; D'Angelo et al. 2020). Furthermore, inflammatory processes, oxidative stress, and stress hormones (e.g., cortisol) are suggested to interact with BDNF (Schaafsma et al. 2017). It has also been proposed that such stressors might play a role in perinatal depression, at least in subgroups (Payne and Maguire 2019). Therefore, various endo- and exogenous stressors may lead to a reduction in BDNF, disturbing neuroplasticity and causing depressive symptoms in mothers (Phillips 2017). However, how infants are potentially affected by dysregulated maternal BDNF remains unknown. The possibility to identify specific treatments to ameliorate the effects of dysregulated maternal BDNF should be investigated in future longitudinal studies, as a potential avenue for treating perinatal depression.

## Supplementary Information

Below is the link to the electronic supplementary material.

Supplementary file1 (DOCX 21 kb)

Supplementary file2 (DOCX 14 kb)

Supplementary file3 (JPG 111 kb)

Supplementary file4 (JPG 109 kb)

Supplementary file5 (JPG 118 kb)

Supplementary file6 (JPG 67 kb)

Supplementary file7 (DOCX 12 kb)

Supplementary file8 (DOCX 13 kb)

## Data Availability

The anonymized raw results can be made available on request.
