# Supplementary material for: A pilot study of multilevel analysis of BDNF in paternal and maternal perinatal depression
Source: Arch Womens Ment Health. 2022 Jan 6;25(1):237–49. doi: 10.1007/s00737-021-01197-2 (PMC8784499; doi:10.1007/s00737-021-01197-2)
Supplement: Supplementary file 1 — Supplementary file1 (DOCX 21 kb) [file 737_2021_1197_MOESM1_ESM.docx]

**Supplementary Material**

**Participants**

The couples were recruited during parent information evenings (Bürgerhospital, Frankfurt). Here, once every month, expecting parents are given information about the Department of Obstetrics at the Bürgerhospital, Frankfurt. The department has >2000 birth per year and besides the Department of obstetrics at the University Hospital Frankfurt, is the largest facility for obstetrics and also has a perinatal center level 1. Around 200 expecting parents and their family members participated (before the Corona pandemic) in the information event per month to decide if they would chose the Bürgerhospital as birth clinic for their child. After the usual information, from July 2017-July 2019, the study personal of the VBS study informed about the study, distributed flyers and collected contact details from the audience. The sample therefore was recruited from a population-based group of expecting parents in Frankfurt and the nearer regions. Furthermore, midwives in Frankfurt were informed about the study and asked to make couples aware of the study. In total, about 2000 couples (excluding other family members) were made aware pf the study. Due to the very broad and naturalistic study in- and exclusion criteria, we did not exclude a couple that was interested in participating in the study at all. Inclusion criteria were woman’s pregnancy at > 20 gestational weeks, capable of giving informed consent, fluent command of the German language, and both expecting parents willing to participate. Exclusion criteria were pregnancy at < 20 gestational weeks, insufficiently fluent German, unable to give informed consent, and only one partner wanting to participate in the study.

However, after the first contact, initially 90 couples were included and had given informed consent, but 4 dropped out without being assessed for the baseline visit due to lack of time, later additional 5 couples dropped out because of incomplete data of the baseline study visit but no further response to us. 81 couples could then be assessed and analysed in the baseline visit.

**Biomaterial sampling**

We obtained blood samples from 87.6 % participants for the first sampling (in pregnancy, T0), 83.0% at 3 month pp and at 58 % 6 months pp. Blood was taken from the participants by venous puncture, usually in the evening. The participants were not fasting, as in an earlier study we found that fasting or non-fasting status did not significantly influence BDNF serum levels (Schroter, Brum et al. 2019). Blood from EDTA-Monovettes was used for DNA isolation, for subsequent genotyping and epigenetic analysis. For peripheral BDNF measurement, serum was separated by centrifugation at 4°C for 10 minutes at 2300 rpm and aliquoted in 500 µl. After that, serum was stored at -80°C until used as previously described (Schroter, Brum et al. 2020).

**Genotyping**

Genomic DNA was obtained from 162 participants (72 mothers, 78 fathers). DNA was isolated from EDTA blood by a standard procedure, as published previously (Miller, Dykes et al. 1988). DNA concentration and quality were assessed by spectrophotometric measurement (Infinite 200 PRO-Tecan). Genotyping of BDNF rs6265 was performed by KASP Assay (He, Holme et al. 2014) according to manufacturer’s instructions (LGC Genomics) (for further details see Supplementary Material). 5 to 50 ng/µl of DNA was tested in a 384-well plate by Wet DNA method. Thermal cycling conditions used are reported in Supplemental table 1. Fluorescent signal from the two FRET cassettes (FAM (T Allel) and HEX (C Allel)) was detected with LightCycler 480 (Roche).

**BDNF gene DNA methylation analysis**

Aliquots of genomic DNA (500 ng) were bisulfite converted using the EpiTect 96 Bisulfite Kit (Qiagen) according to manufacturer’s instructions. Two amplicons covering sections of BDNF exon I promoter were amplified by PCR using the following oligonucleotides, which were specifically designed to amplify bisulfite converted DNA sequences using the PyroMark Assay Design 2.0 Software (Qiagen) (for details see Supplementary Material).

**Serum BDNF levels**

ELISA (double antibody sandwich enzyme-linked immunosorbent assay; DBD00, R&D Systems, USA) was used to measure BDNF protein concentration in serum. Protocols were conducted according to the manufacturer’s instructions and as described in our previous work (Schroter, Brum et al. 2019). In detail, serum was diluted 1:20 with H20. 50 µl of each sample or standard was combined with 100 µl diluent and added to each well, and conjugated to a monoclonal antibody specific for free human BDNF. The plate was incubated for 2 hours. Next, 100 µl of this solution was conjugated to a horseradish peroxidase conjugated secondary antibody and preservatives added, and the plate was incubated for 1 hour at room temperature. The plate was subsequently washed a total of 3 times. Colour reagents containing stabilized hydrogen peroxide and stabilized chromogen (tetramethylbenzidine) were then added and incubated for 30 minutes. Finally, the reaction was stopped by adding sulfuric acid, producing a colour change from blue to yellow. The absorption was measured at 450 nm within 30 minutes with wavelength correction set to 540 nm (Tecan plate reader). The range of detection was from 62.5 – 4000 pg/mL.

**Limitations**

The results of our pilot study should be interpreted in the context of several limitations. Firstly, the number of participants with depressive symptoms was relatively small, and most individuals were only mildly depressed. Secondly, although the time of blood sampling was mostly in the afternoons, this was not standardized due to organisation issues. The participants were therefore not fasting, which could have confounded the results, although in our previous study we found that fasting status did not significantly influence BDNF serum concentration (Schroter, Brum et al. 2019). Thirdly, there are several extraneous variables that could influence the risk of perinatal depression (such as pregnancy/birth complications and traumatic experience of the birth) which we were unable to explore in this study. Fourthly, we did not collect data regarding whether participants were receiving treatment for depression. Finally, we did not assess blood cell counts to correct for cell type differences in the methylation analysis.

**Conclusion**

In conclusion, we found similar-to-higher prevalence rates of perinatal depression in mothers and fathers compared to previous studies, and could also confirm several psychosocial risk factors for developing perinatal depression. Alterations to BDNF at the epigenetic and protein level seem to play a role in maternal perinatal depression, but not paternal. To further clarify the role of BDNF in perinatal depression, future studies should be performed using larger cohorts and participants with more severe depression, and include data regarding potential confounding variables such as physical activity, sex and stress hormones.
