# Supplementary material for: A pilot study of multilevel analysis of BDNF in paternal and maternal perinatal depression
Source: Arch Womens Ment Health. 2022 Jan 6;25(1):237–49. doi: 10.1007/s00737-021-01197-2 (PMC8784499; doi:10.1007/s00737-021-01197-2)
Supplement: Supplementary file 2 — Supplementary file2 (DOCX 14 kb) [file 737_2021_1197_MOESM2_ESM.docx]

Supplemental Figures Legends

**Figure 1:** Depressive symptoms from pregnancy to 12 month postpartum (EPDS)

Mean Scores +/- standard deviation of the Edinburgh Postnatal Depression scale (EPDS) are shown. Statistical differences between men and women were calculated by Mann-Whitney-U test. Level of significance was set at p≤0.05. *p≤0.05, **p≤0.01

**Figure 2:** Depressive symptoms from pregnancy to 12 month postpartum (MADRS total score)

Mean Scores +/- standard deviation of the Montgomery Ǻsberg Depression Rating Scale (MADRS) are shown. Statistical differences between men and women were calculated by Mann-Whitney-U test. Level of significance was set at p≤0.05. *p≤0.05, **p≤0.01

**Figure 3:** Depressive symptoms from pregnancy to 12 month postpartum (MADRS without sleep item)

Mean Scores +/- standard deviation of the Montgomery Ǻsberg Depression Rating Scale (MADRS) without sleep item are shown. Statistical differences between men and women were calculated by Mann-Whitney-U test. Level of significance was set at p≤0.05. *p≤0.05, **p≤0.01

**Figure 5:** BDNF protein level

Mean levels of BDNF serum concentration +/- standard deviation are shown. Pregnant women had significantly lower levels than men (Mann-Whitney-U-Test, p<0.0001), which then were significantly higher after 3 month postpartum compared with the fathers (Mann-Whitney-U-Test, p=0.024). No significant gender difference was seen at 6 months postpartum (Mann-Whitney-U-Test, p=0.156). Level of significance was set at p≤0.05. * p≤0.05; ***p≤0.001
