# Supplementary figures and images for: A pilot study of multilevel analysis of BDNF in paternal and maternal perinatal depression
Source: Arch Womens Ment Health. 2022 Jan 6;25(1):237–49. doi: 10.1007/s00737-021-01197-2 (PMC8784499; doi:10.1007/s00737-021-01197-2)

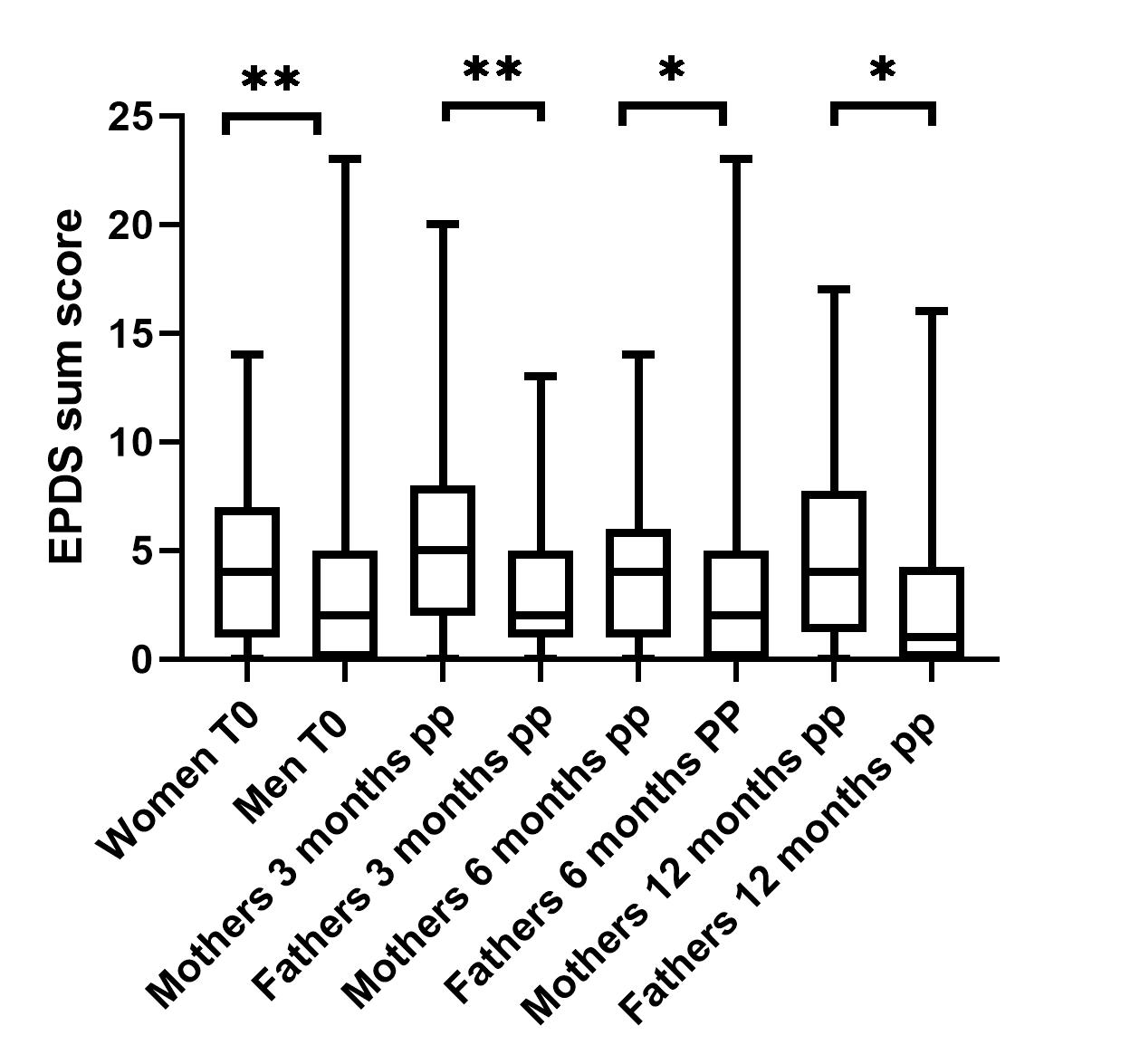

Supplement: Supplementary file 3 — Supplementary file3 (JPG 111 kb) [file 737_2021_1197_MOESM3_ESM.jpg]

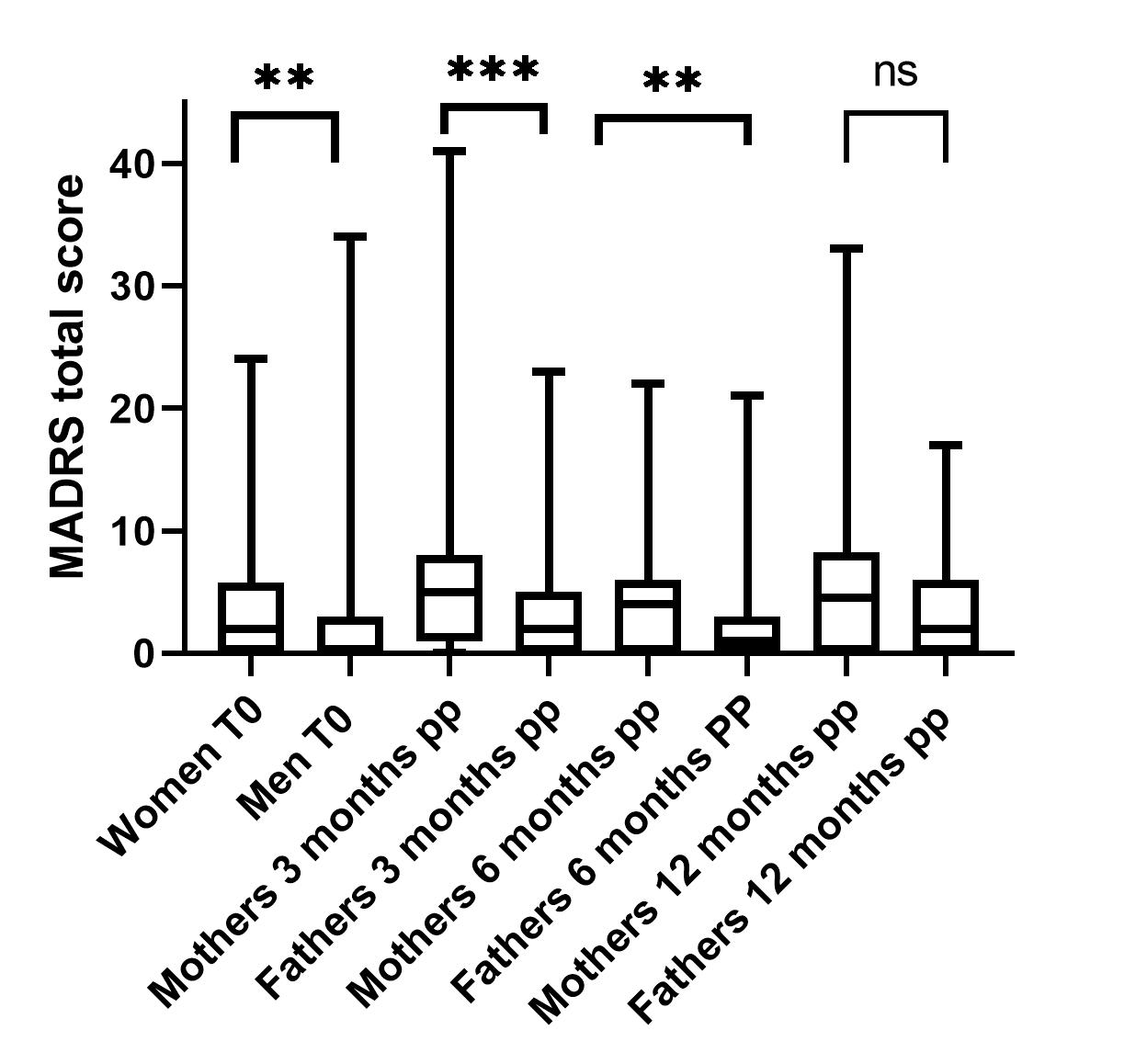

Supplement: Supplementary file 4 — Supplementary file4 (JPG 109 kb) [file 737_2021_1197_MOESM4_ESM.jpg]

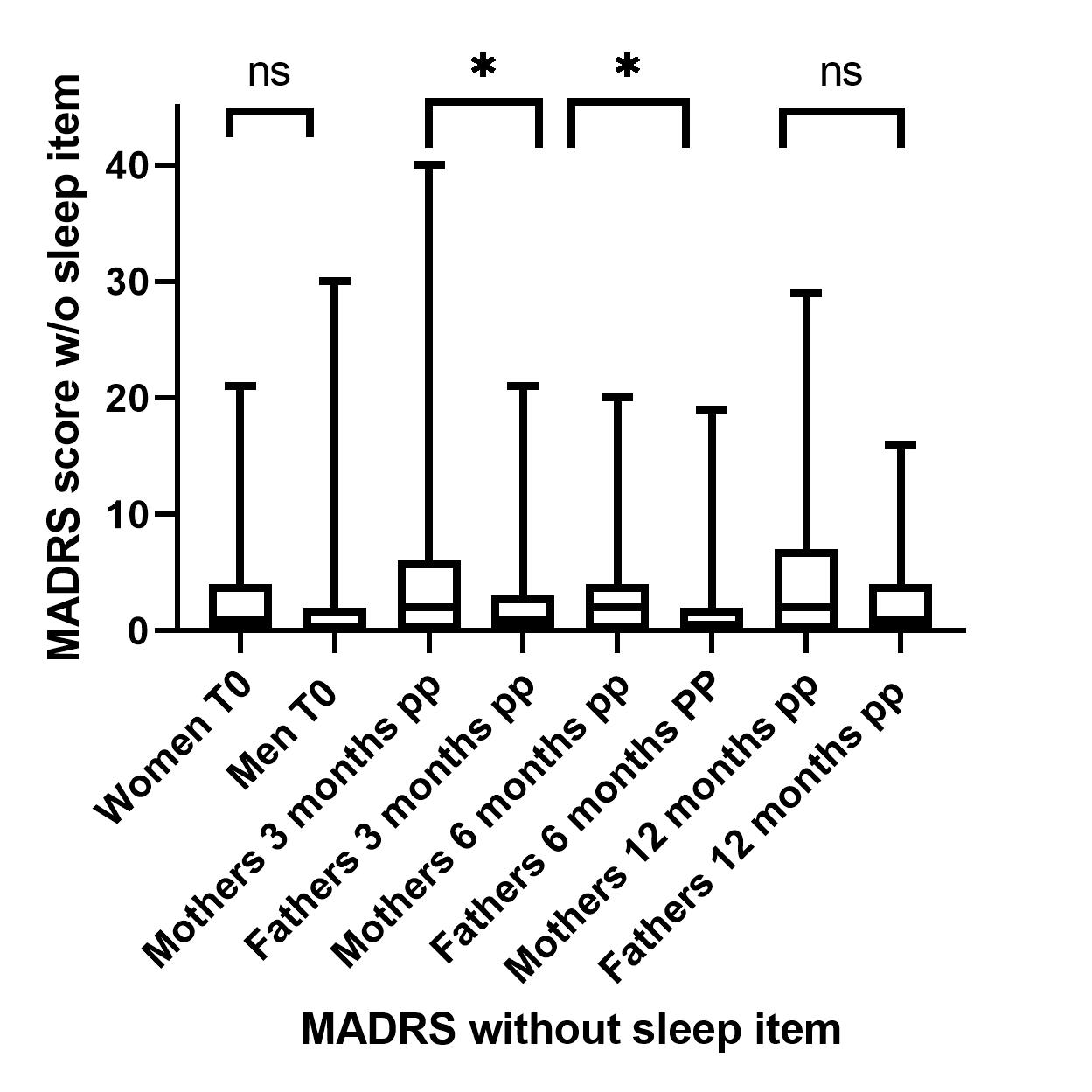

Supplement: Supplementary file 5 — Supplementary file5 (JPG 118 kb) [file 737_2021_1197_MOESM5_ESM.jpg]

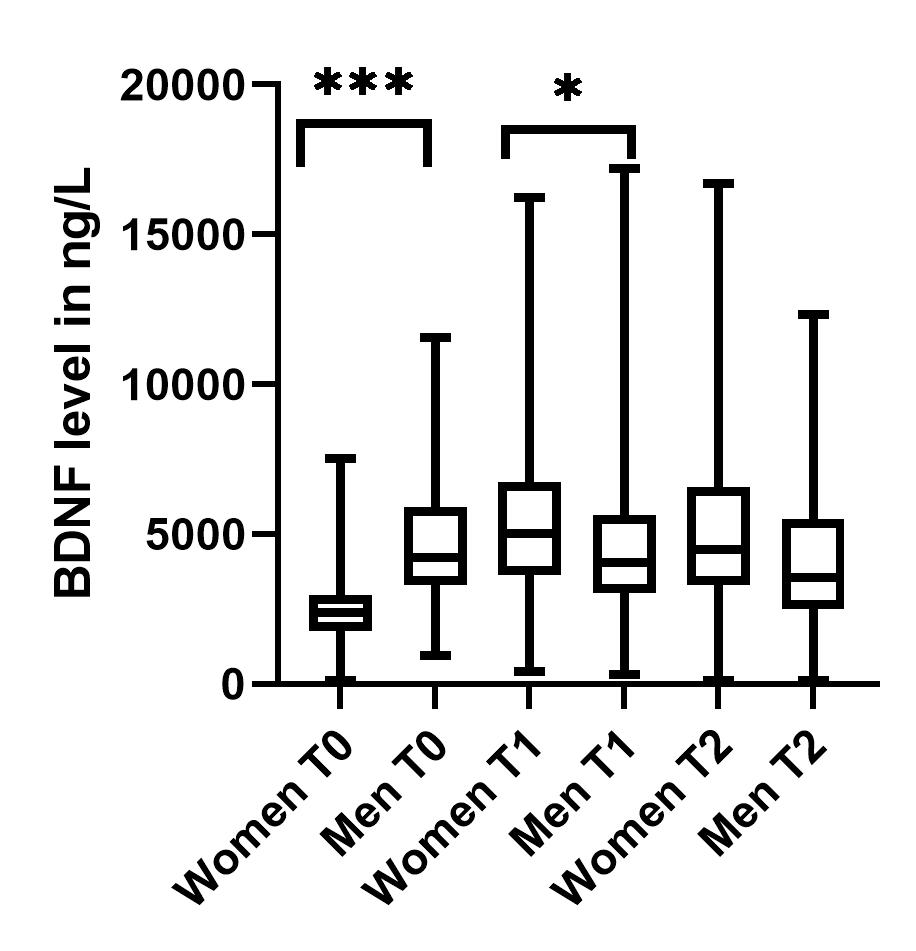

Supplement: Supplementary file 6 — Supplementary file6 (JPG 67 kb) [file 737_2021_1197_MOESM6_ESM.jpg]
