# Supplementary material for: A pilot study of multilevel analysis of BDNF in paternal and maternal perinatal depression
Source: Arch Womens Ment Health. 2022 Jan 6;25(1):237–49. doi: 10.1007/s00737-021-01197-2 (PMC8784499; doi:10.1007/s00737-021-01197-2)
Supplement: Supplementary file 7 — Supplementary file7 (DOCX 12 kb) [file 737_2021_1197_MOESM7_ESM.docx]

**Supplemental Table 1**: Severity of depressive symptoms (MADRS total score)

|  | **Men**  **(n=81)** | | | **Women**  **(n=81)** | | |
| --- | --- | --- | --- | --- | --- | --- |
| **Severity of depression** | **mild**  N (%) | **moderate**  N (%) | **severe**  N (%) | **mild**  n (%) | **moderate**  n (%) | **severe**  N (%) |
| **Pregnancy**  **(n=81)** | 5 (6.2%) | 2 (2.5%) | 0 (0%) | 15 (18.5%) | 1 (1.23 %) | 0 (0%) |
| **3 months pp**  **(n=73)** | 10 (13.7%) | 2 (2.7%) | 0 (0%) | 25 (34.3%) | 2 (2.7%) | 1 (1.4%) |
| **6 months pp**  **(n=60)** | 8 (13.3%) | 1 (1.67%) | 0 (0%) | 9 (15 %) | 2 (3.33%) | 0 (0%) |
| **12 months pp**  **(n=51)** | 9 (17.64%) | 0 (0%) | 0 (0%) | 14 (27.45%) | 1 (1.96%) | 0 (0%) |

The severity of depression was rated using the Montgomery Ǻsberg Depression Scale (MADRS): Sum score 0 - 6 no depression, 7 - 19 mild depression, 20 - 34 moderate depression, 35 - 60 severe depression. Percentage of the whole sample, separated into females and males, is given.
