# Supplementary material for: A pilot study of multilevel analysis of BDNF in paternal and maternal perinatal depression
Source: Arch Womens Ment Health. 2022 Jan 6;25(1):237–49. doi: 10.1007/s00737-021-01197-2 (PMC8784499; doi:10.1007/s00737-021-01197-2)
Supplement: Supplementary file 8 — Supplementary file8 (DOCX 13 kb) [file 737_2021_1197_MOESM8_ESM.docx]

**Supplemental Table 2**: Severity of depressive symptoms (MADRS without sleep item)

|  | **Men** | | | **Women**  **(n=81)** | | |
| --- | --- | --- | --- | --- | --- | --- |
| **Severity of depression** | **mild**  N (%) | **moderate**  N (%) | **severe**  N (%) | **mild**  n (%) | **moderate**  n (%) | **severe**  N (%) |
| **Pregnancy (n=81)** | 4 (5%) | 2 (2.5%) | 0 (0%) | 11 (13.58%) | 0 (0 %) | 0 (0%) |
| **3 months pp**  **(n=73)** | 11 (15.1%) | 1 (1.37%) | 0 (0%) | 12 (16.4%) | 2 (2.7%) | 1 (1.4%) |
| **6 months pp**  **(n=60)** | 7 (11.67%) | 0 (0%) | 0 (0%) | 6 (10.0 %) | 1 (1.6%) | 0 (0%) |
| **12 months pp**  **(n=51)** | 7 (13.7%) | 0 (0%) | 0 (0%) | 11 (21.6%) | 1 (1.96%) | 0 (0%) |

The severity of depression was rated by using the Montgomery Ǻsberg Depression Scale (MADRS) without the sleep item: Sum score 0 - 6 no depression, 7 - 19 mild depression, 20 - 34 moderate depression, 35 - 60 severe depression. Percentage of the whole sample, separated into females and males, is given.
